# Supplementary material for: Radical Stress Is More Cytotoxic in the Nucleus than in Other Organelles
Source: Int J Mol Sci. 2019 Aug 25;20(17):4147. doi: 10.3390/ijms20174147 (PMC6747261; doi:10.3390/ijms20174147)
Supplement: Supplementary file 1 [file ijms-20-04147-s001.zip › Supplementary Material_sequences.pdf]

Sequences

SuperNova-myc-N1

ATGGGCAGCGAGGTGGGCCCCGCCCTGTTCCAGAGCGACATGACCTTCAAGATCTTCATCGACGGCGAGGTGAAC
GGCCAGAAGTTCACCATCGTGGCCGACGGCAGCAGCAAGTTCCCCCACGGCGACTTCAACGTGCACGCCGTGTGC
GAGACCGGCAAGCTGCCCATGAGCTGGAAGCCCATCTGCCACCTGATCCAGTACGGCGAGCCCTTCTTCGCCCGC
TACCCCGACGGCATCAGCCACTTCGCCCAGGAGTGCTTCCCCGAGGGCCTGAGCATCGACCGCACCGTGCGCTTC
GAGAACGACGGCACCATGACCAGCCACCACACCTACGAGCTGGACGACACCTGCGTGGTGAGCCGCATCACCGTG
AACTGCGACGGCTTCCAGCCCGACGGCCCCATCATGCGCGACCAGCTGGTGGACATCCTGCCCAGCGAGACCCAC
ATGTTCCCCCACGGCCCCAACGCCGTGCGCCAGACCGCCACCATCGGCTTCACCACCGCCGACGGCGGCAAGATG
ATGGGCCACTTCGACAGCAAGATGACCTTCAACGGCAGCCGCGCCATCGAGATCCCCGGCCCCCACTTCGTGACC
ATCATCACCAAGCAGACCCGCGACACCAGCGACAAGCGCGACCACGTGTGCCAGCGCGAGGTGGCCTACGCCCAC
AGCGTGCCCCGCATCACCAGCGCCATCGGCAGCGACGAGGACGAGCAGAAGCTGATCAGCGAGGAGGACCTGTAA

COX8-SuperNova

ATGAGCGTGCTGACCCCCCTGCTGCTGCGCGGCCTGACCGGCAGCGCCCGCCGCCTGCCCGTGCCCCGCGCCAAG
ATCCACAGCCTGAAGCTTCGAATTCTGCAGTCGACGGTACCGCGGGCCCGGGATCCACCCGTGGCCACCATGGGC
AGCGAGGTGGGCCCCGCCCTGTTCCAGAGCGACATGACCTTCAAGATCTTCATCGACGGCGAGGTGAACGGCCAG
AAGTTCACCATCGTGGCCGACGGCAGCAGCAAGTTCCCCCACGGCGACTTCAACGTGCACGCCGTGTGCGAGACC
GGCAAGCTGCCCATGAGCTGGAAGCCCATCTGCCACCTGATCCAGTACGGCGAGCCCTTCTTCGCCCGCTACCCC
GACGGCATCAGCCACTTCGCCCAGGAGTGCTTCCCCGAGGGCCTGAGCATCGACCGCACCGTGCGCTTCGAGAAC
GACGGCACCATGACCAGCCACCACACCTACGAGCTGGACGACACCTGCGTGGTGAGCCGCATCACCGTGAACTGC
GACGGCTTCCAGCCCGACGGCCCCATCATGCGCGACCAGCTGGTGGACATCCTGCCCAGCGAGACCCACATGTTC
CCCCACGGCCCCAACGCCGTGCGCCAGACCGCCACCATCGGCTTCACCACCGCCGACGGCGGCAAGATGATGGGC
CACTTCGACAGCAAGATGACCTTCAACGGCAGCCGCGCCATCGAGATCCCCGGCCCCCACTTCGTGACCATCATC
ACCAAGCAGACCCGCGACACCAGCGACAAGCGCGACCACGTGTGCCAGCGCGAGGTGGCCTACGCCCACAGCGTG
CCCCGCATCACCAGCGCCATCGGCAGCGACGAGGACGAGCAGAAGCTGATCAGCGAGGAGGACCTGTAA

VAMP8-SuperNova

ATGGAGGAGGCCAGTGGGAGTGCCGGAAATGACCGAGTTAGGAACCTGCAGAGTGAGGTGGAGGGAGTCAAGAAT
ATTATGACCCAGAATGTGGAGCGGATCTTGTCCAGAGGGGAGAACCTGGACCACCTCCGAAACAAGACAGAGGAC
TTGGAAGCCACGTCTGAACACTTCAAGACAACGTCCCAGAAGGTGGCCCGGAAGTTCTGGTGGAAGAATGTGAAG
ATGATTGTCATCATCTGTGTGATTGTCCTTATCATCGTCATCCTCATTATACTTTTTGCCACTGGTACCATCCCC
ACTAAGGATCCACCCGTGGCCACCATGGGCAGCGAGGTGGGCCCCGCCCTGTTCCAGAGCGACATGACCTTCAAG
ATCTTCATCGACGGCGAGGTGAACGGCCAGAAGTTCACCATCGTGGCCGACGGCAGCAGCAAGTTCCCCCACGGC
GACTTCAACGTGCACGCCGTGTGCGAGACCGGCAAGCTGCCCATGAGCTGGAAGCCCATCTGCCACCTGATCCAG
TACGGCGAGCCCTTCTTCGCCCGCTACCCCGACGGCATCAGCCACTTCGCCCAGGAGTGCTTCCCCGAGGGCCTG
AGCATCGACCGCACCGTGCGCTTCGAGAACGACGGCACCATGACCAGCCACCACACCTACGAGCTGGACGACACC
TGCGTGGTGAGCCGCATCACCGTGAACTGCGACGGCTTCCAGCCCGACGGCCCCATCATGCGCGACCAGCTGGTG
GACATCCTGCCCAGCGAGACCCACATGTTCCCCCACGGCCCCAACGCCGTGCGCCAGACCGCCACCATCGGCTTC
ACCACCGCCGACGGCGGCAAGATGATGGGCCACTTCGACAGCAAGATGACCTTCAACGGCAGCCGCGCCATCGAG
ATCCCCGGCCCCCACTTCGTGACCATCATCACCAAGCAGACCCGCGACACCAGCGACAAGCGCGACCACGTGTGC
CAGCGCGAGGTGGCCTACGCCCACAGCGTGCCCCGCATCACCAGCGCCATCGGCAGCGACGAGGACGAGCAGAAG
CTGATCAGCGAGGAGGACCTGTAA

SuperNova-TGON2

ATGGTAAAGCTTGGTTCAGAGGTGGGCCCCGCCCTGTTCCAGAGCGACATGACCTTCAAAATCTTCATCGACGGC
GAGGTGAACGGCCAGAAGTTCACCATCGTGGCCGACGGCAGCAGCAAGTTCCCCCACGGCGACTTCAACGTGCAC
GCCGTGTGCGAGACCGGCAAGCTGCCCATGAGCTGGAAGCCCATCTGCCACCTGATCCAGTACGGCGAGCCCTTC
TTCGCCCGCTACCCCGACGGCATCAGCCATTTCGCCCAGGAGTGCTTCCCCGAGGGCCTGAGCATCGACCGCACC
GTGCGCTTCGAGAACGACGGCACCATGACCAGCCACCACACCTACGAGCTGGACGACACCTGCGTGGTGAGCCGC
ATCACCGTGAACTGCGACGGCTTCCAGCCCGACGGCCCCATCATGCGCGACCAGCTGGTGGACATCCTGCCCAGC
GAGACCCACATGTTCCCCCACGGCCCCAACGCCGTGCGCCAGACCGCCACCATCGGCTTCACCACCGCCGACGGC
GGCAAGATGATGGGCCACTTCGACAGCAAGATGACCTTCAACGGCAGCCGCGCCATCGAGATCCCCGGCCCACAC
TTCGTGACCATCATCACCAAGCAGACCAGGGACACCAGCGACAAGCGCGACCACGTGTGCCAGCGCGAGGTGGCC
TACGCCCACAGCGTGCCCCGCATCACCAGCGCCATCGGTAGCGACGAGGATTCGAATTCTATGCAGTTCCTGGTG
GCCCTGCTGCTGCTGAGCGTGGCCGTGGCCCGCGCCCTGCCCAGCGCCAGCAAGCCCAACAACACCAGCAGCGAG
AACAACCCCCCCATCCAGCCCAGCACCCCCCTGCCCCCCGGCGTGGACATCAGCCAGCAGGTGAAGACCAACCGC
CCCACCGACCAGCGCCTGGAGAGCGACAAGGAGGGCCAGGACAAGACCGTGGCCCGCACCAGCGCCAGCGTGAGC
AGCGGCGTGGAGAGCGCCACCAACCTGAACCTGGACGACAGCAAGAAGCACCCCGAGACCGCCGACGCCAAGCTG
AAGGAGACCCTGCAGCAGCTGCTGCCCGTGGACCCCAAGCAGGAGAAGAGCGGCCAGAAGTTCACCAAGGACAGC
GGCAGCCCCACCGGCGGCGACAGCGACAACACCACCGGCGGCGACAGCAACAAGACCACCGGCGTGGACAGCGAC
AAGACCAGCGGCGGCGACAGCAACAAGCCCACCGGCAGCGACAACGACAAGCCCACCGGCGGCGACAGCAACAAG
CCCACCAGCAAGGTGCCCAGCAACACCGAGACCCCCAAGATCGACAAGGTGCAGCTGACCGAGAAGGGCCAGAAG
CCCACCCTGATCAGCAAGACCGAGAGCGGCGAGAAGCTGGCCGGCGACAGCGACTTCAGCCTGAAGCCCGAGAAG
GGCGACAAGAGCAGCGAGCCCACCGAGGACGTGGAGACCAAGGAGATCGAGGAGGGCGACACCGAGCCCGAGGAG
GGCAGCCCCCTGGAGGAGGAGAACGAGAAGGTGCTGGGCCCCAGCAGCAGCGAGAACCAGGAGGGCACCCTGACC
GACAGCATGAAGGACGAGAAGGACGACCACTACAAGGACAACAGCGGCAACACCAGCGCCGAGAGCAGCCACTTC
TTCGCCTACCTGGTGACCGCCGCCGTGCTGGTGGCCGTGCTGTACATCGCCTACCACAACAAGCGCAAGATCATC
GCCTTCGCCCTGGAGGGCAAGCGCAGCAAGGTGACCCGCCGCCCCAAGGCCAGCGACTACCAGCGCCTGAACCTG
AAGCTGCGGGATCCACCCGGGATCTAG

ER-SuperNova

ATGGATCTGCTGAGCGTGCCCCTGCTGCTGGGCCTGCTGGGCCTGGCCGTGGCCCGGGATCCAGGTTCAGAGGTG
GGCCCCGCCCTGTTCCAGAGCGACATGACCTTCAAAATCTTCATCGACGGCGAGGTGAACGGCCAGAAGTTCACC
ATCGTGGCCGACGGCAGCAGCAAGTTCCCCCACGGCGACTTCAACGTGCACGCCGTGTGCGAGACCGGCAAGCTG
CCCATGAGCTGGAAGCCCATCTGCCACCTGATCCAGTACGGCGAGCCCTTCTTCGCCCGCTACCCCGACGGCATC
AGCCATTTCGCCCAGGAGTGCTTCCCCGAGGGCCTGAGCATCGACCGCACCGTGCGCTTCGAGAACGACGGCACC
ATGACCAGCCACCACACCTACGAGCTGGACGACACCTGCGTGGTGAGCCGCATCACCGTGAACTGCGACGGCTTC
CAGCCCGACGGCCCCATCATGCGCGACCAGCTGGTGGACATCCTGCCCAGCGAGACCCACATGTTCCCCCACGGC
CCCAACGCCGTGCGCCAGACCGCCACCATCGGCTTCACCACCGCCGACGGCGGCAAGATGATGGGCCACTTCGAC
AGCAAGATGACCTTCAACGGCAGCCGCGCCATCGAGATCCCCGGCCCACACTTCGTGACCATCATCACCAAGCAG
ACCAGGGACACCAGCGACAAGCGCGACCACGTGTGCCAGCGCGAGGTGGCCTACGCCCACAGCGTGCCCCGCATC
ACCAGCGCCATCGGTAGCGACGAGGATCTCGAGACCGGTAAGGACGAGCTGTAA

NLS-SuperNova

ATGCCCGCCGCCAAGCGCGTGAAGCTGGACGTGGATCCAGGCAGCGAGGTGGGCCCCGCCCTGTTCCAGAGCGAC
ATGACCTTCAAGATCTTCATCGACGGCGAGGTGAACGGCCAGAAGTTCACCATCGTGGCCGACGGCAGCAGCAAG
TTCCCCCACGGCGACTTCAACGTGCACGCCGTGTGCGAGACCGGCAAGCTGCCCATGAGCTGGAAGCCCATCTGC
CACCTGATCCAGTACGGCGAGCCCTTCTTCGCCCGCTACCCCGACGGCATCAGCCACTTCGCCCAGGAGTGCTTC
CCCGAGGGCCTGAGCATCGACCGCACCGTGCGCTTCGAGAACGACGGCACCATGACCAGCCACCACACCTACGAG
CTGGACGACACCTGCGTGGTGAGCCGCATCACCGTGAACTGCGACGGCTTCCAGCCCGACGGCCCCATCATGCGC
GACCAGCTGGTGGACATCCTGCCCAGCGAGACCCACATGTTCCCCCACGGCCCCAACGCCGTGCGCCAGACCGCC
ACCATCGGCTTCACCACCGCCGACGGCGGCAAGATGATGGGCCACTTCGACAGCAAGATGACCTTCAACGGCAGC
CGCGCCATCGAGATCCCCGGCCCCCACTTCGTGACCATCATCACCAAGCAGACCCGCGACACCAGCGACAAGCGC
GACCACGTGTGCCAGCGCGAGGTGGCCTACGCCCACAGCGTGCCCCGCATCACCAGCGCCATCGGCAGCGACGAG
GACGAGCAGAAGCTGATCAGCGAGGAGGACCTGTAA
