# Supplementary figures and images for: Radical Stress Is More Cytotoxic in the Nucleus than in Other Organelles
Source: Int J Mol Sci. 2019 Aug 25;20(17):4147. doi: 10.3390/ijms20174147 (PMC6747261; doi:10.3390/ijms20174147)

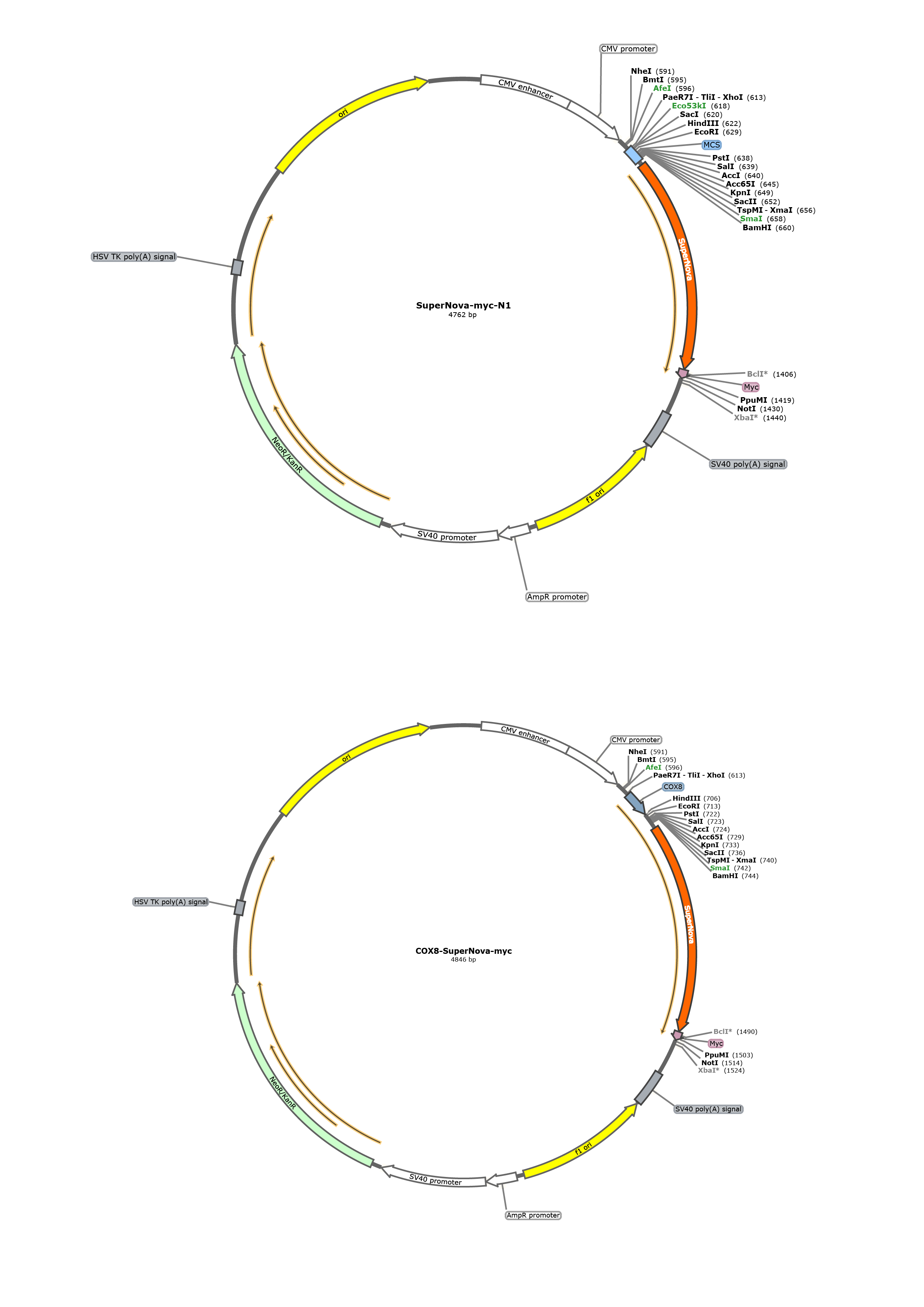

Supplement: Supplementary file 1 [file ijms-20-04147-s001.zip › Sup Fig 1 plasmid maps-01.png]

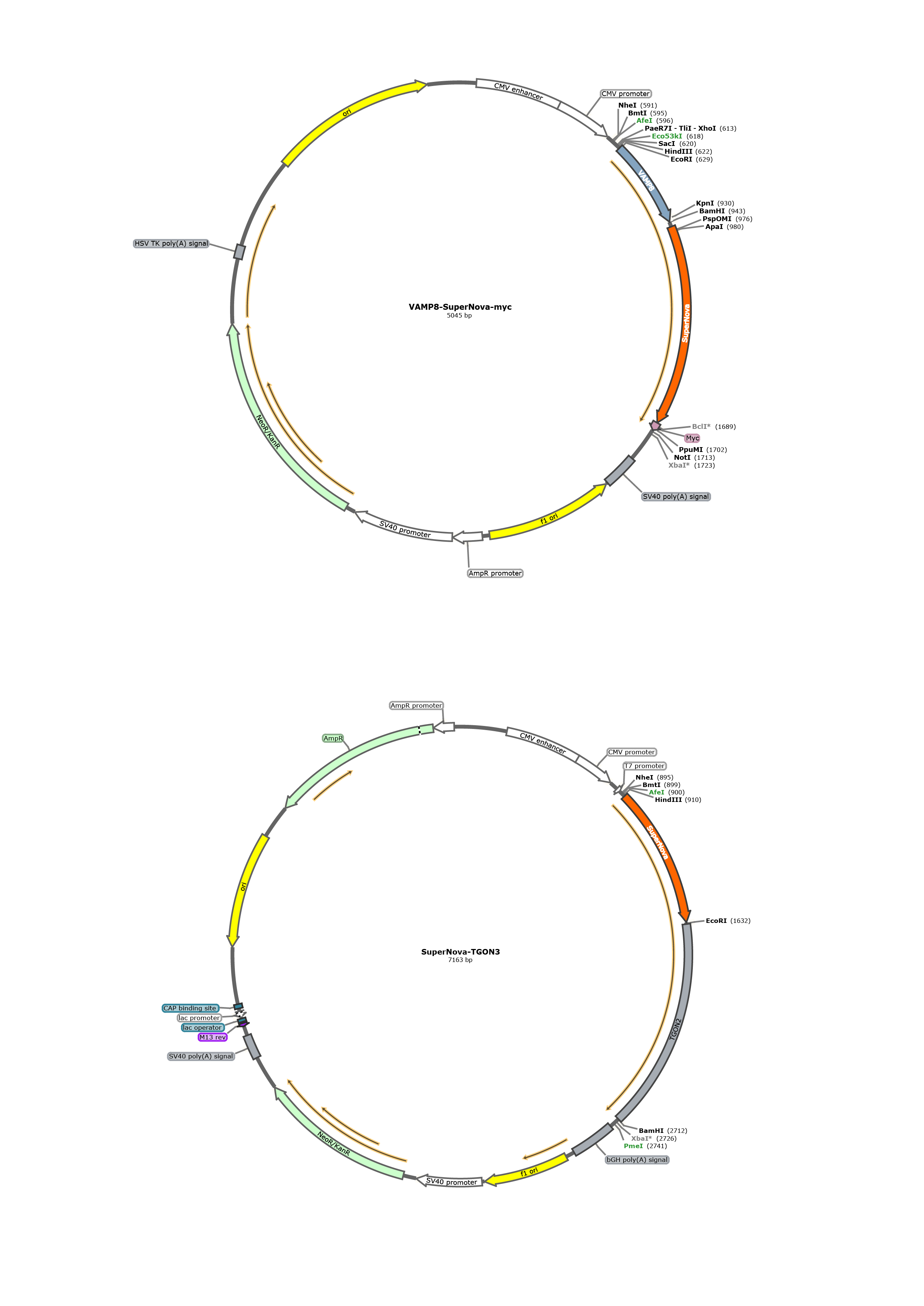

Supplement: Supplementary file 1 [file ijms-20-04147-s001.zip › Sup Fig 1 plasmid maps-02.png]

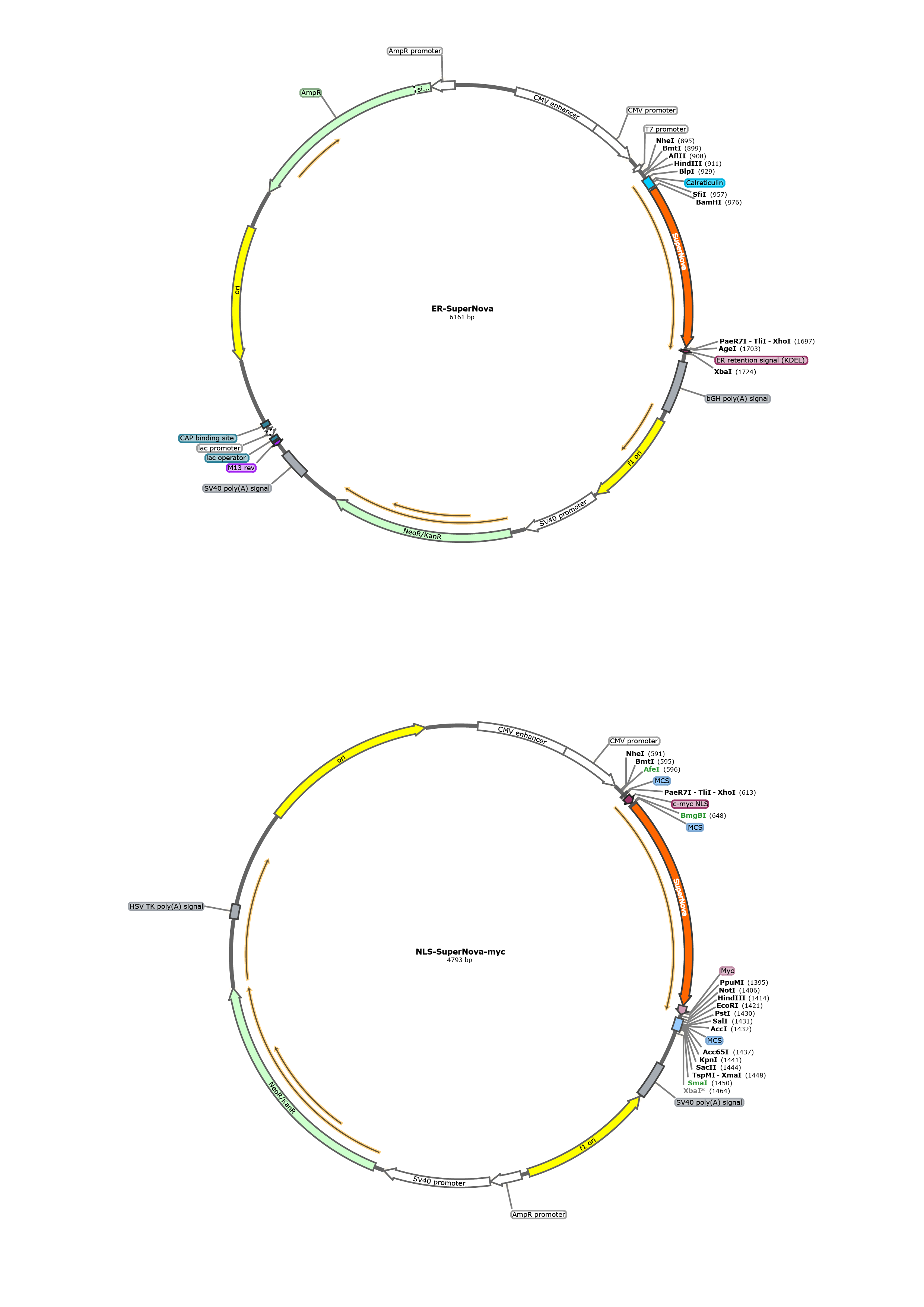

Supplement: Supplementary file 1 [file ijms-20-04147-s001.zip › Sup Fig 1 plasmid maps-03.png]
